# Trend, determinants, and future prospect of child marriage in the Amhara region, Ethiopia: a multivariate decomposition analysis

**DOI:** 10.3389/fpubh.2023.1132148

**Published:** 2023-09-14

**Authors:** Anteneh Mengist Dessie, Denekew Tenaw Anley, Melkamu Aderajew Zemene, Natnael Atnafu Gebeyehu, Getachew Asmare Adella, Gizachew Ambaw Kassie, Misganaw Asmamaw Mengstie, Mohammed Abdu Seid, Endeshaw Chekol Abebe, Molalegn Mesele Gesese, Kirubel Dagnaw Tegegne, Rahel Mulatie Anteneh, Yenealem Solomon, Natnael Moges, Berihun Bantie, Sefineh Fenta Feleke, Tadesse Asmamaw Dejenie, Habtamu Geremew

**Affiliations:** ^1^Department of Public Health, College of Health Sciences, Debre Tabor University, Debre Tabor, Ethiopia; ^2^Department of Midwifery, College of Medicine and Health Science, Wolaita Sodo University, Wolaita Sodo, Ethiopia; ^3^Department of Reproductive Health and Nutrition, School of Public Health, Woliata Sodo University, Woliata Sodo, Ethiopia; ^4^Department of Epidemiology and Biostatistics, School of Public Health, Woliata Sodo University, Woliata Sodo, Ethiopia; ^5^Department of Biochemistry, College of Health Sciences, Debre Tabor University, Debre Tabor, Ethiopia; ^6^Unit of Physiology, Department of Biomedical Science, College of Health Science, Debre Tabor University, Debre Tabor, Ethiopia; ^7^Department of Nursing, College of Medicine and Health Science, Wollo University, Dessie, Ethiopia; ^8^Department of Medical Laboratory Science, College of Health Sciences, Debre Tabor University, Debre Tabor, Ethiopia; ^9^Department of Pediatrics and Child Health Nursing, College of Health Sciences, Debre Tabor University, Debre Tabor, Ethiopia; ^10^Department of Comprehensive Nursing, College of Health Sciences, Debre Tabor University, Debre Tabor, Ethiopia; ^11^Department of Public Health, College of Health Sciences, Woldia University, Woldia, Ethiopia; ^12^Department of Medical Biochemistry, College of Medicine and Health Sciences, University of Gondar, Gondar, Ethiopia; ^13^College of Health Science, Oda Bultum University, Chiro, Ethiopia

**Keywords:** child marriage, trend, future prospect, multivariate decomposition, Amhara region

## Abstract

**Background:**

Child marriage is a harmful traditional practice, which compromises children of their childhood and threatens their lives and health. In Ethiopia, 58% of women and 9% of men get married before the age of 18 years. Surprisingly, parents in the Amhara region make marriage promises of their children before they are even born, which will hinder the region from attaining the Sustainable Development Goal of ending child marriage. Thus, this study aimed to assess the trends, determinants, and future prospects of child marriage in the Amhara region of Ethiopia.

**Methods:**

A repeated cross-sectional study was conducted using four consecutive nationally representative Ethiopian demographic and health surveys (2000–2016). A logit-based multivariate decomposition analysis for a non-linear response model was fitted to identify factors that contributed to the change in child marriage over time. Statistical significance was declared at a *p*-value of < 0.05. The child marriage practice in the Amhara region by the year 2030 was also predicted using different forecasting features of Excel.

**Results:**

The trend of child marriage over the study period (2000–2016) decreased from 79.9% (76.7, 82.8) to 42.9% (39.1, 46.9), with an annual average reduction rate of 2.9%. Approximately 35.2% of the decline resulted from an increase in the proportion of women who attained secondary and above-secondary education over the two surveys. A decrease in the proportion of rural women and a change in the behavior of educated and media-exposed women also contributed significantly to the decline in child marriage. The prevalence of child marriage in the Amhara region by the year 2030 was also predicted to be 10.1% or 8.8%.

**Conclusion:**

Though there has been a significant decline in child marriage in the Amhara region over the past 16 years, the proportion is still high, and the region is not going to eliminate it by 2030. Education, residence, and media exposure were all factors associated with the observed change in child marriage in this study. Therefore, additional efforts will be required if child marriage is to be eliminated by 2030, and investing more in education and media access will hasten the region's progress in this direction.

## Introduction

Child marriage is defined as any formal marriage or informal union between a child below the age of 18 years and an adult or another child (1). Child marriage is known to be practiced globally, affecting both boys and girls. However, girls are disproportionately more affected (1, 2), and South Asia and Sub-Saharan Africa (SSA) account for 44% and 18% of all child marriages worldwide, respectively (3). In Ethiopia, 58% of women and 9% of men get married before the age of 18 years (4). Surprisingly, some parents in the Amhara region pledge to offer their child in marriage before the child is even born, which makes the region have the highest rate of child marriage among all the regions of the country (4, 5).

Child marriage substantially affects women's experiences of pregnancy and childbirth and the health of the women and their offspring. Prolonged labor, abortion, hemorrhage during birth, spousal violence, pressure or stress, and termination of education were identified among women who get married early (6–8). Child marriage also has a negative attribution to cervical cancer and sexually transmitted infections (STIs) due to the inability to negotiate safer sex (9, 10). In general, the World Bank outlined that by 2030, four trillion dollars will be lost due to child marriage (2). Studies from different continents indicated that educational status, residence, income level, employment status, family size, knowledge about legal marital age, and parental decision-making about when to get married were the most important determinants of child marriage (8, 11–14).

The United Nations Sustainable Development Goal (SDG) 5 target 3 aspires to eliminate child marriage by 2030 (15). In line with this, the Ethiopian government has taken various legal, institutional, and strategic measures to end child marriage. Recently, the government has committed to ending child marriage by 2025 and has established a National Alliance to End Child Marriage. The Ethiopian Ministry of Children, Women, and Youth Affairs is in charge of this national alliance. The Ethiopian Ministry of Women, Child, and Youth Affairs also formulated the National Strategy and Action Plan on Harmful Traditional Practices against Women and Children in 2013 (16–18). However, although there has been a reduction in the rate of child marriage from 49% in 2005 to 40% in 2016 in Ethiopia (19), it is still a major public health threat affecting the lives of many children, particularly in the Amhara region, where 73% of women aged 15–49 years were married before the age of 18 years (11).

In addition to being a violation of human rights, child marriage also prevents many other development goals from being reached. Without considerable progress on ending child marriage, nearly half of the SDGs—including those that address poverty, health, education, nutrition, food security, economic growth, and inequality reduction—will not be met (20). When girls are married off early, they are prevented from attending school, forced to have children before they are ready, and subjected to abuse and exploitation. These could delay the global advancements related to gender equality in education and health.

However, although there is evidence regarding the burden and determinants of child marriage, there is a dearth of information regarding the trend, determinants, and future prospects of child marriage in the Amhara region, which is a major contributor to the highest child marriage rate in Ethiopia. Knowing the factors associated with prevalence and trends in child marriage in the Amhara region of Ethiopia including a forecast of future prevalence is crucial to tailoring our actions toward ending this harmful practice. Hence, this study aimed to determine the trend, determinants, and future prospects of child marriage in the Amhara region of Ethiopia.

## Methods and materials

### Study design, area, and period

The nationally representative repeated cross-sectional study design was employed using the four consecutive Ethiopian Demographic and Health Survey (EDHS) datasets (EDHS 2000, EDHS 2005, EDHS 2011, and EDHS 2016). The Amhara region in which this study was conducted is a regional state located in the northwestern part of Ethiopia between 8°45′ and 13°45′ north latitude and 36° 20′ and 40° 20′ east longitude. Amhara is subdivided into 13 administrative zones (Wag Himra, North Wollo, North Gondar, Central Gondar, West Gondar, South Gondar, South Wollo, North Shewa, Oromia, East Gojjam, West Gojjam, Awi, and Bahir Dar special zone), and its land area is estimated at ~1,70,000 square km.

### Data sources, study populations, and sampling

**Figure 1:**
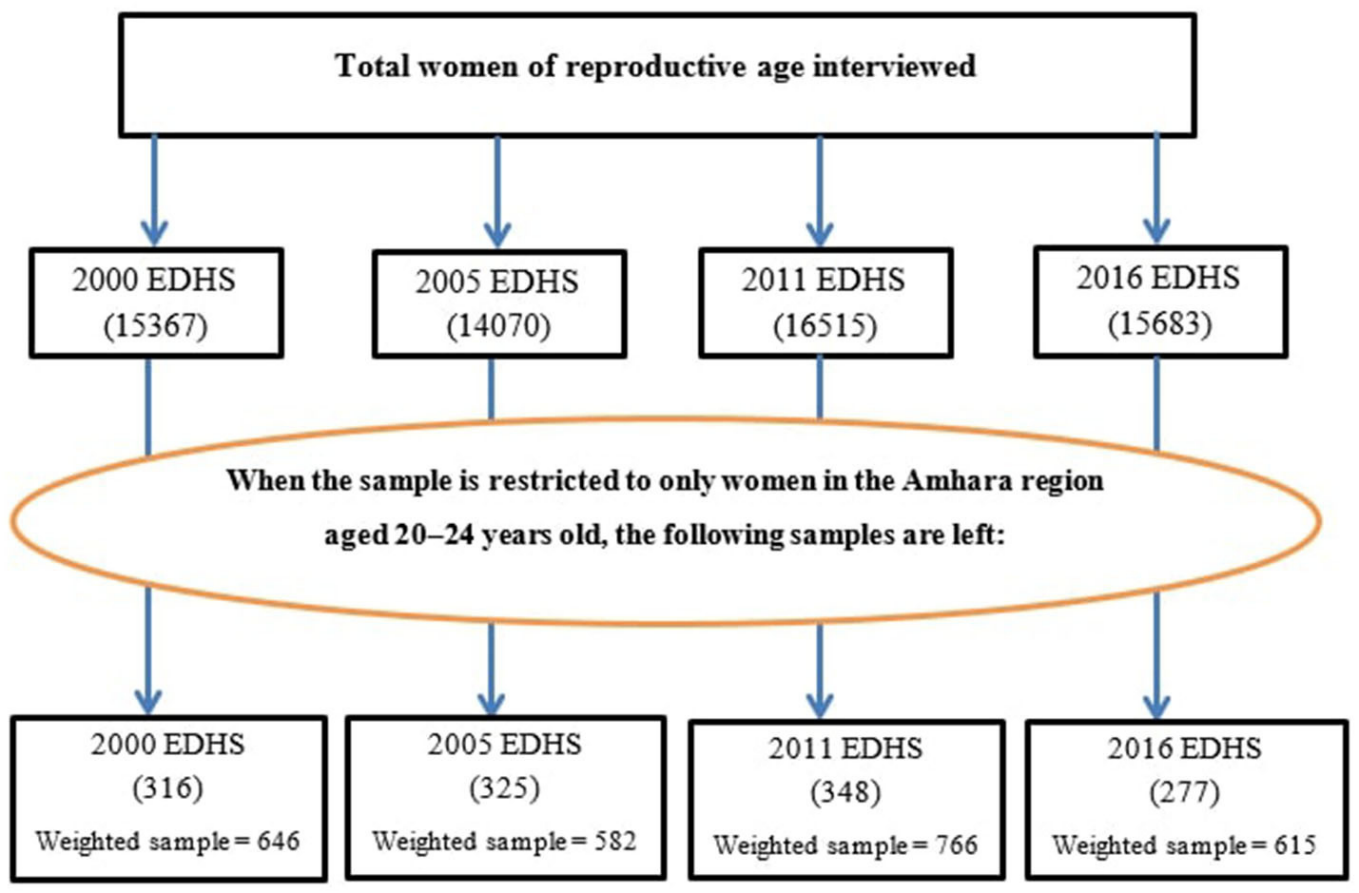
Detail procedure of the sample size and sampling technique used to reach at the final sample size.

The data for this study were accessed and downloaded from the webpage of the International Demographic and Health Survey (DHS) Program (http://www.dhsprogram.com) after subscribing as an authorized user. The DHS collects data through nationally representative cross-sectional surveys to generate updated health and health-related indicators. Ethiopia has undertaken four consecutive major DHS surveys in 2000, 2005, 2011, and 2016. EDHS uses a two-stage stratified cluster sampling technique. Detailed sampling procedures are available in the full EDHS reports (4). However, to assess the prevalence of child marriage, this study used the SDG indicator 5.3.1—the percentage of women aged 20 to 24 years who were first married or in a union before the age of 18 years. Accordingly, a total weighted sample of 646, 582, 766, and 615 women aged 20–24 were used from the 2000, 2005, 2011, and 2016 EDHSs, respectively (Figure 1).

### Variables of the study

The outcome variable of this study was child marriage. It is a binary response variable that asks whether women marry or be in a union before the age of 18 years and receives a “yes” or “no” response. This variable was constructed using marital status and age at first marriage/cohabitation variables available in the datasets. All background characteristics of the women including residence, educational status, religion, sex of the household head, age, marital status, family size, media exposure, working status, and relation to the household head were taken as potential predictor variables for the four consecutive surveys.

### Data management and analysis

The data were managed and analyzed using STATA version 16/MP and Microsoft Excel software as appropriate. The overall analysis in this study was carried out on the weighted samples. A descriptive analysis was performed to observe the trends with a 95% confidence interval (CI) of child marriage in the four survey years. The trends were explored separately for the periods 2000–2005, 2005–2011, 2011–2016, and 2000–2016. In relation to this, the determination of the future prospect of child marriage by 2030 was performed using the forecast functions of Excel. Both linear regression forecasting and exponential smoothing forecasts were done. Linear regression forecasting uses linear regression, while exponential smoothing forecasting uses the exponential triple smoothing (ETS) algorithm, which smooths out minor deviations in past data trends by detecting seasonality patterns and confidence intervals.

Then, a non-linear multivariate logit decomposition model was implemented to determine the extent to which factors contributed to the observed change in child marriage among women aged 20–24 years. This statistical approach uses the output from regression models and divides the difference in a statistic, such as a mean or proportion, between the two points in time into two parts. The first component is attributable to the variety in the survey population's structure, differences in characteristics (endowments “E”), and the second is attributable to the change in the survey participants' behavior and differences in coefficients (coefficients “C”). The dependent variable is the function of the linear combination of predictors and regression coefficients:


$$\begin{array}{l} \left(\text{Y}=\text{F }\left(\text{Xβ}\right)\right), \end{array}$$

where

Y is the N × 1 dependent variable vector, X is an N × K matrix of independent variables, and β is a K × 1 vector of coefficients.

The proportion difference in Y between survey A and survey B of successive EDHS surveys can be decomposed as Y_A_-Y_B_ = F (X_A_β_A_) – F (X_B_β_B_).

For the log odds of home delivery, the proportion of the model is written as


$$\begin{array}{l} \text{Logit }\left({\text{Y}}_{\text{A}}\right)-\text{logit }\left({\text{Y}}_{\text{B}}\right)=\text{F}\left({\text{X}}_{\text{A}}{\text{β}}_{\text{A}}\right)-\text{F }\left({\text{X}}_{\text{B}}{\text{β}}_{\text{B}}\right)\\ =\underset{\text{E}}{\underset{︸}{\text{F}\left({\text{X}}_{\text{A}}{\text{β}}_{\text{A}}\right)-\text{F}\left({\text{X}}_{\text{B}}{\text{β}}_{\text{A}}\right)}}+\underset{\text{C}}{\underset{︸}{\text{F}\left({\text{X}}_{\text{B}}{\text{β}}_{\text{A}}\right)-\text{F }\left({\text{X}}_{\text{B}}{\text{β}}_{\text{B}}\right)}} \end{array}$$

## Results

### Background characteristics of the study population

This section presents the background characteristics of respondents over four consecutive EDHS surveys (EDHS 2000, 2005, 2011, and 2016). In all surveys, the mean age of respondents was almost exactly 21.69 (±1.46 SD). There was a slight increment in urban residents from 11.2% in 2000 to 18.7% in 2016. Notable progress has been observed in terms of education. More than half of women have no formal education in the first three consecutive surveys (80.1% in 2000, 76.3% in 2005, and 51.4% in 2011), whereas only 30.5% of women were uneducated in the EDHS 2016. Religion, mean family size, and wealth index did not show a valuable change in the composition of women across the surveys (Table 1).

**Table 1:** Percentage distribution of background characteristics of young women aged 20–24 years in 2000, 2005, 2011, and 2016 Ethiopian demographic and health surveys.

**Variables**	**Category**	**Weighted frequency (%)**			
		**EDHS 2000 (*****N*** = **646)**	**EDHS 2005 (*****N*** = **582)**	**EDHS 2011 (*****N*** = **766)**	**EDHS 2016 (*****N*** = **615)**
Age	Mean ± SD	21.7 ± 1.5	21.6 ± 1.5	21.7 ± 1.5	21.7 ± 1.4
Residence	Urban	72 (11.2)	81 (14.0)	178 (23.2)	115 (18.7)
	Rural	574 (88.8)	501 (86.0)	588 (76.8)	500 (81.3)
Marital status	Currently married	445 (68.8)	414 (71.2)	466 (60.8)	348 (56.6)
	Formerly married	128 (19.8)	90 (15.5)	115 (15.1)	80 (13.0)
	Never married	73 (11.4)	78 (13.3)	185 (24.1)	187 (30.4)
Religion	Orthodox	530 (82.0)	475 (81.6)	659 (86.1)	533 (86.7)
	Muslim	114 (17.7)	99 (17.0)	107 (13.9)	80 (13.0)
	Others^*^	2 (0.3)	8 (1.4)	0 (0.0)	2 (0.3)
Occupation	Working	450 (69.6)	157 (27.0)	231 (30.0)	191 (31.0)
	Not working	196 (30.4)	425 (73.0)	535 (70.0)	425 (69.0)
Educational status	No education	517 (80.1)	444 (76.3)	394 (51.4)	187 (30.5)
	Primary	76 (11.7)	66 (11.4)	210 (27.4)	221 (35.9)
	Secondary	53 (8.2)	62 (10.6)	73 (9.5)	128 (20.8)
	Higher	0 (0.0%)	10 (1.7)	89 (11.7)	79 (12.8)
Family size	≤ 5	488 (75.6)	457 (78.5)	562 (73.3)	474 (77.0)
	>5	158 (24.4)	125 (21.5)	204 (26.7)	141 (23.0)
Wealth index	Poor	–	190 (32.6)	288 (37.6)	203 (33.0)
	Middle	–	151 (26.0)	166 (21.7)	128 (20.8)
	Rich	–	241 (41.4)	312 (40.7)	284 (46.2)
HH head	Male	525 (81.2)	480 (82.6)	540 (70.5)	462 (75.1)
	Female	121 (18.8)	102 (17.4)	226 (29.5)	153 (24.9)
Relation to HH head	Head	49 (7.6)	33 (5.7)	101 (13)	63 (10.2)
	Wife	400 (61.9)	355 (61.1)	349 (45.6)	274 (44.6)
	Daughter	141 (21.9)	134 (22.9)	190 (24.7)	211 (34.3)
	Others^**^	56 (8.6)	60 (10.3)	126 (16.4)	67 (10.9)
Media exposure	Yes	57 (8.9)	112 (19.3)	257 (33.6)	124 (20.1)
	No	589 (91.1)	470 (80.7)	509 (66.4)	491 (79.9)

^*^protestant/others; HH, Household; ^**^daughter-in-law/grand-daughter/sister/other relative/adopted/not related.

### Trend and future prospect of child marriage in the Amhara region

**Figure 2:**
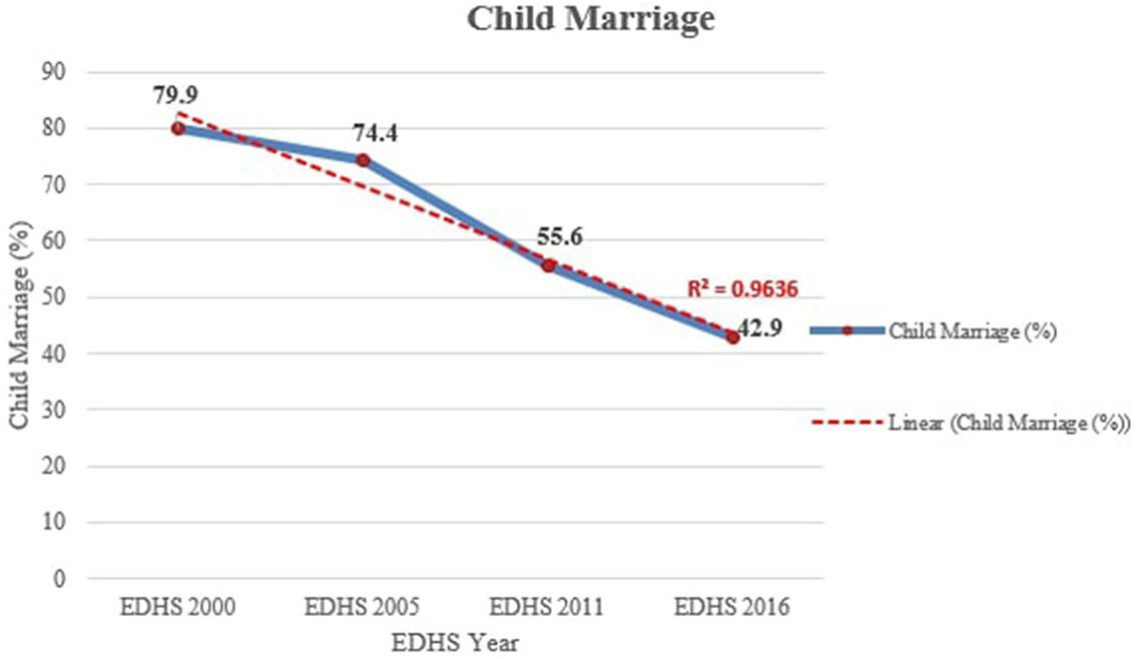
Trend of child marriage in Amhara region, Ethiopia from 2000 to 2016.

The time period was separated into four sections, 2000–2005, 2005–2011, 2011–2016, and 2000–2016, in order to see the trend in the prevalence of child marriage over time. The trend of child marriage over the study period (2000–2016) showed a significant decline, which decreased from 79.9% (76.7, 82.8) to 42.9% (39.1, 46.9) with an annual average reduction rate of 2.9%. The largest decline was observed in the survey period 2005–2011 with an 18.8% drop down from 74.4% (70.7, 77.8) to 55.6% (52.0, 59.1) and in the survey period 2011–2016 with a 12.7% decrement from 55.6% (52.0, 59.1) to 42.9% (39.1, 46.9), respectively. The trend was shown to be closer to the fitted linear regression line, as shown by the coefficient of determination (R-squared) value. A linear model explained 96.4% of the response variable variation (Figure 2). Under 15 years, marriage also showed a greater decline from 50.2% (46.3, 54.0) in 2000 to 16.3% (13.6, 19.5) in 2016. Among the 428 young women who reported practicing marriage in the 2016 survey, 71.3% of them got married by their parents' decision, and 33.9% of women were students before marriage.

The trend of child marriage over the study period (2000–2016) varied by the background characteristics of women in the four surveys. There was a decrease in child marriage proportion among all categories of residence with a higher decrease among rural residents from 84.4% in 2000 to 50.2% in 2016. Similar results were seen for the prevalence of child marriage, which decrease from 82.7% to 47.2% among women who had no media exposure and from 52.3% to 26.2% among those who had.

**Figure 3:**
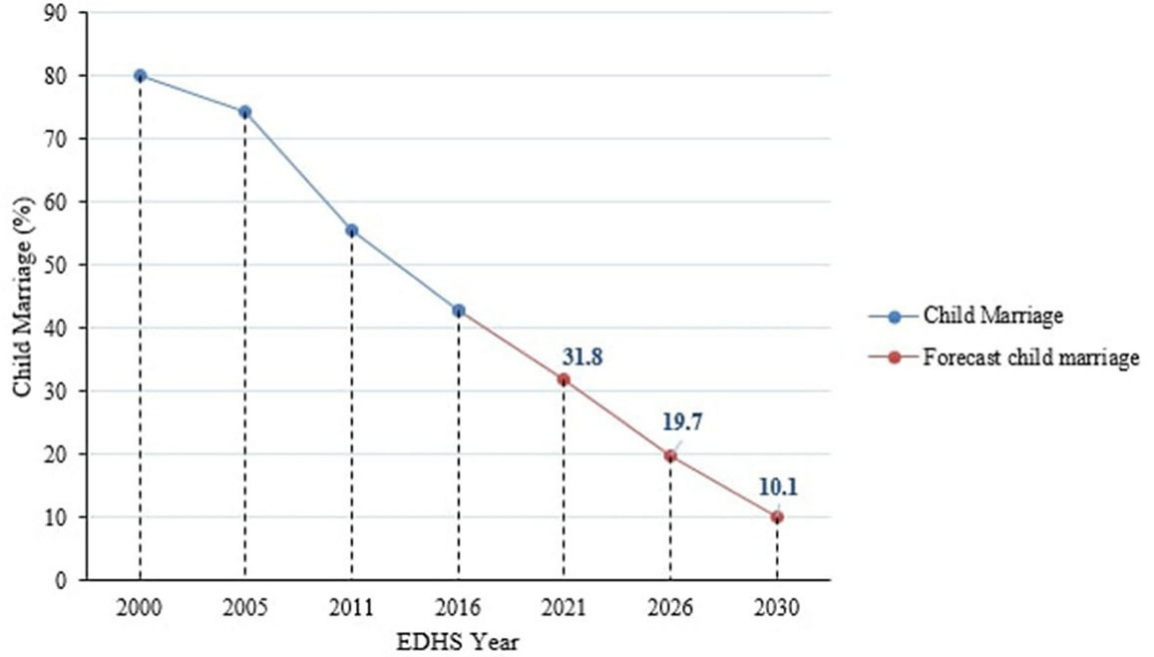
Forecasting the future prospect of child marriage in Amhara region using FORECAST.LINEAR function of excel.

**Figure 4:**
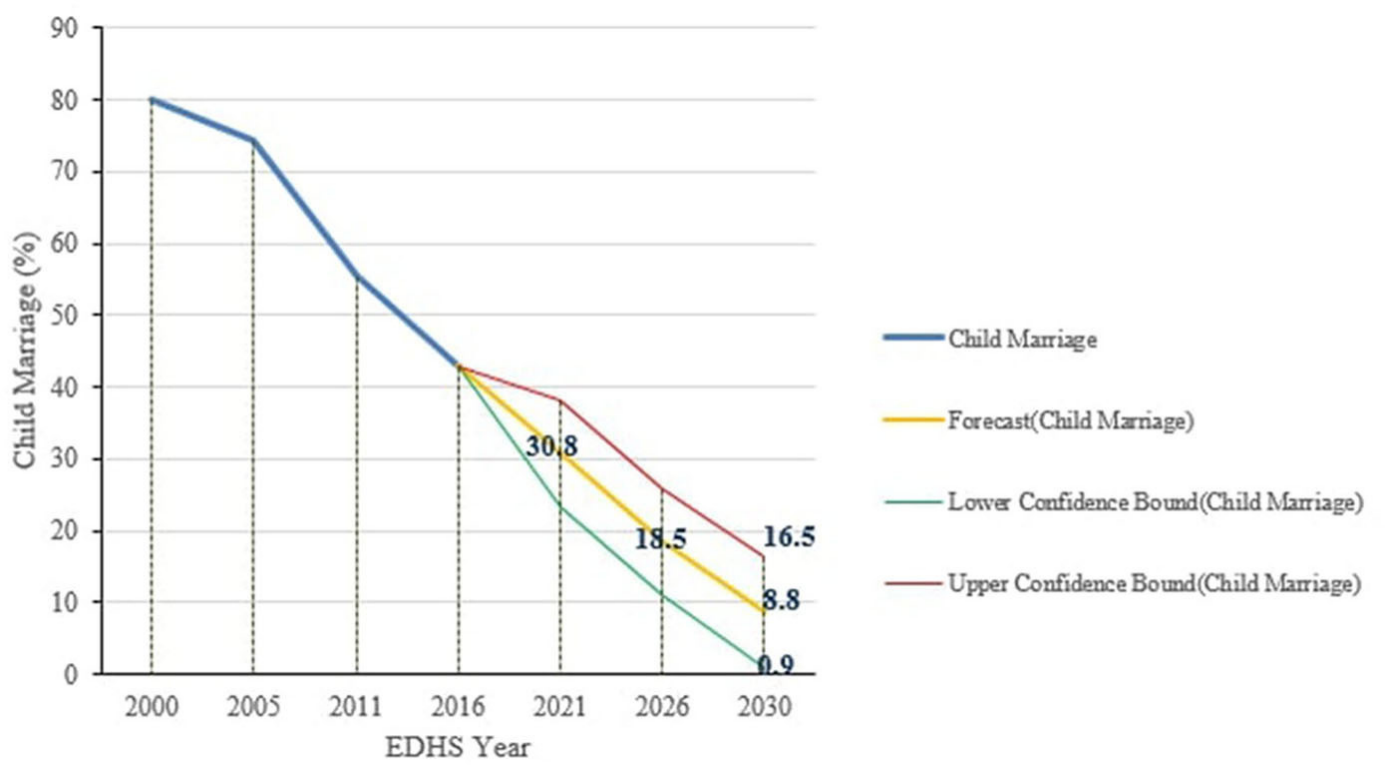
Forecasting the future prospect of child marriage in Amhara region using FORECAST.ETS function of excel.

Using Excel's forecasting features, child marriage in the Amhara region by the year 2030 was predicted. Linear regression forecasting uses linear regression, while exponential smoothing forecasting uses the ETS algorithm, which smooths out minor deviations in past data trends by detecting seasonality patterns and confidence intervals. As a result, the prevalence of child marriage by 2030 was predicted to be 10.1% and 8.8% (0.9, 16.5) in linear regression and exponential smoothing forecasts, respectively (Figures 3, 4).

### Factors associated with a change in child marriage

#### Decomposition analysis

The practice of child marriage changed significantly by 37 percentage points from 2000 to 2016. The multivariate decomposition logistic regression analysis revealed that approximately 50.2% of this change in child marriage was due to differences in compositional characteristics (endowments). After controlling for the compositional change, 49.8% of the change in child marriage was due to the differences in the effects of specific characteristics (coefficient) rather than the structural composition of women in the two surveys.

Among the compositional change factors, education level and residence had a statistically significant effect on the change contribution. The increase in the proportion of women who attain secondary and above education from 8.2% in 2000 to 33.6% in 2016 had an important compositional contribution to the decline of child marriage by 35.2%. The decrement in the composition of rural women in the sample also showed a significant positive impact on the decline of child marriage practice.

Educational status and media exposure had statistically significant effects on the coefficient contribution to the observed change in child marriage in the Amhara region, Ethiopia. Controlling all compositional change factors, ~6.7% and 2.0% of the decrement in child marriage over the last decade resulted from changes in the behavior of women who attained secondary and above education and have media exposure, respectively (Table 2).

**Table 2:** Detail decomposition analysis of early marriage in the Amhara region from 2000 to 2016.

**Variables**	**Difference due to characteristics (E)**		**Difference due to coefficient (C)**	
	**Coef. (95% CI)**	**Pct. (%)**	**Coef. (95% CI)**	**Pct. (%)**
**Residence**				
Urban Rural	1 −0.0197 (−0.0408, −0.0013)^*^	1 5.342	1 0.0544 (−0.1795, 0.2882)	1 −14.695
**Educational status**				
No education Primary Secondary and above	1 −0.0287 (−0.0607,0.0033) −0.1302 (−0.1710, −0.0894)^*^	1 7.766 35.204	1 −0.0059 (−0.0284,0.0166) −0.0246 (−0.0449, −0.0044)^*^	1 1.598 6.663
**Religion**				
Orthodox Muslim^**+**^	1 0.0022 (−0.0052,0.0096)	1 −0.593	1 −0.0219 (−0.0553,0.0115)	1 5.920
**Family size**				
≤ 5 >5	1 0.0007 (−0.0018, 0.0032)	1 −0.192	1 −0.0170 (−0.0623, 0.0282)	1 4.608
**Sex of HH head**				
Male Female	1 0.0028 (−0.0083, 0.0138)	1 −0.752	1 0.0084 (−0.0294, 0.0462)	1 −2.266
**Relation to HH head**				
Head Wife Daughter Others^++^	1 −0.0104 (−0.0626, 0.0417) −0.0257 (−0.0571, 0.0057) −0.0029 (−0.0090, 0.0031)	1 2.824 6.9480.790	1 0.0325 (-0.1728,0.2379) 0.0263 (−0.0379, 0.0906) 0.0158 (−0.0098, 0.0414)	1 −8.794 −7.114 −4.260
**Media exposure**				
Yes No	0.0054 (-0.0181,0.0289) 1	−1.454 1	−0.0072 (-0.0173, -0.0016)^*^ 1	1.939 1
**Working status**				
Working Not working	0.0210 (−0.0307, 0.0727) 1	−5.673 1	−0.0786 (−0.1824, 0.0252) 1	21.253 1
Constant			−0.1805 (−0.5561,0.1950)	44.921
Overall	−0.1857 (−0.2776, −0.0938)^*^	50.212	−0.1842 (−0.2968, −0.0715)^*^	49.788

^+^Four protestants/others; ^++^daughter-in-law/grand-daughter/sister/other relative/adopted/not related.

^*^p < 0.05; HH, household; CI, confidence interval; Pct., percentage contribution; Coef., coefficient; 1, reference.

## Discussion

Despite the world's promises to end child marriage, many children continue to get married early (before the age of 18 years), which compromises their childhood and threatens their lives and health (21). Therefore, this study was to examine the trend and future prospects of child marriage in the Amhara region and identify significant determinants that contributed to the change in child marriage practice during the past 16 years in the Amhara region, Ethiopia.

The results of this study showed that child marriage prevalence decreased significantly from 79.9% to 42.9% over the study period (2000–2016), which was lowered by 46.3% with an annual reduction rate of 2.9%. This finding is consistent with the drop in child marriage prevalence in Sub-Saharan countries (22), whereas the annual reduction rate of child marriage found in this study was higher than the global annual reduction rate of 1.9% over the past 10 years, which may be attributable to Ethiopia's strong policy and legal frameworks, such as the National Alliance to End Child Marriage by 2025 (23). Contrary to what was found in this study, child marriage prevalence in eastern Ethiopia increased at an annual rate of 2.9% between 2008 and 2018, rising from 32.4% to 40.2% (24). This might be due to the fact that countries will have different age requirements for marriage depending on religious affiliation, and some cultures will allow marriage under the age of 18 (25).

If the decline seen in the past 16 years continues, the prevalence of child marriage in the Amhara region of Ethiopia would drop to 10.1% or 8.8% by the year 2030. This is higher than the SDG that aimed to eliminate all harmful practices, including child marriage, by 2030 and Ethiopia's policy to end child marriage by 2025. As a result, if child marriage is to be eliminated in Ethiopia's Amhara region by 2030, additional efforts will be required, and the region should accelerate by setting an annual reduction rate of 7.15%. However, an 11.12% yearly rate of decline would be necessary to end child marriage by 2025, as per Ethiopia's commitment.

The decomposition analysis revealed that both composition (endowments) changes and behavior (coefficient) changes have made almost equal and significant contributions to the decline of child marriage prevalence in the Amhara region over the past 16 years. While the difference in composition (endowment) is responsible for 50.2% of the decrease in child marriage, the difference in characteristics (coefficient) is responsible for ~49.8% of the decline.

Approximately 35.2% of the decrease in child marriage was due to an increase in the proportion of women who attain secondary and above education over the two surveys. After controlling the effects of endowment characteristics, ~6.7% of child marriage reduction was contributed by changes in the behavior of women who attain secondary and above education. This was supported by studies from Bangladesh (26) and Indonesia (27), which reported that education is a strong protective factor against child marriage. The possible justification might be that educated women develop skills, knowledge, and confidence to make informed decisions about when to marry, and being in school might support the idea that girls are still children and too young to marry.

The decrement in the composition of rural women in the sample also showed a significant positive impact on the decline of child marriage practice by 5.3%. This finding is in line with a previous study in Ethiopia (14, 28). A study from SSA regions also revealed that early marriage among rural women was higher compared to that of urban women (12). This may be due to rural areas' higher levels of poverty, stronger social networks, poorer educational prospects, and greater adherence to traditional norms, which may all be directly related to the practice of child marriage. It could also be because women in rural areas are less likely to be aware of the negative effects of early marriage on their health, education, and economy. Additionally, when their parents or guardians violate their human rights, they are unsure of what to do.

After controlling the effects of endowment characteristics, ~2.0% of the reduction in child marriage was due to changes in the behavior of women who had media exposure. Studies from SSA (29) and Asia (30) provided support for this. This might be because the media informs people about child marriage (its consequences and related laws). Films and documentaries, for example, can challenge the perception that getting married after 15 years is not early. In addition, the media adds a human face to the issue. In a way that research and facts are unable to do, hearing and understanding things from the perspective of a young girl fosters empathy. The community that is exposed to the media, as a result, will be informed and modify their behavior in regard to the age at first marriage (31).

This study was based on the Ethiopian demographic health surveys, which are the largest nationally representative datasets. However, the study might have had recall bias since the participants were asked about events that took place 5 years preceding the survey, and the cross-sectional nature of the EDHS data does not allow us to confirm the cause-and-effect relationship. Another limitation of this study is that it only focused on a single regional state of the country. Though the focused regional state was the major contributor to child marriage across the country, it may be more important to undertake a study at the country level and then discuss the regional state variations.

## Conclusion

A significant decline in child marriage has occurred in the Amhara region over the past 16 years. However, the burden is still very high, and the region is not on its way to ending child marriage by 2030. An increase in the proportion of educated women, a decrease in the proportion of rural residents, and the behavioral change of educated and media-exposed women had a significant contribution to the observed decline in child marriage practice. Thus, if child marriage is to be eliminated by the year 2030 in the region, additional efforts will be required from multiple sectors. Investing more in education and media access will accelerate the region's progress toward ending child marriage. The regional health bureau, for instance, should work in collaboration with the education sector and invest in education to send all the girls to school during their school age. Furthermore, scholars are recommended to carry out nationwide community-based implementation research in near future.

## Data availability statement

The datasets presented in this study are publicly available in online repositories from www.measuredhs.com.

## Ethics statement

Ethical approval and informed consent were not required for this specific study since it was a secondary data analysis of publicly available survey data from the Measure DHS program. We requested DHS Programme data archivists, and permission be granted to download and use the data for this study from www.measuredhs.com. All the procedures were carried out in accordance with the relevant regulations and the Declaration of Helsinki.

## Author contributions

All authors made substantial contributions to conception and design, managing the data, or analysis and interpretation of data, took part in drafting the article or revising it critically for important intellectual content, agreed to submit it to the current journal, gave final approval of the version to be published, and agree to be accountable for all aspects of the work.
